# YOLO-GML: An object edge enhancement detection model for UAV aerial images in complex environments

**DOI:** 10.1371/journal.pone.0328070

**Published:** 2025-07-10

**Authors:** Zhihao Zheng, Jianguang Zhao, Jingjing Fan

**Affiliations:** School of Information Engineering, Hebei University of Architecture, Zhangjiakou, China; Shandong Agricultural University, CHINA

## Abstract

Uav target detection is a key technology in low altitude security, disaster relief and other fields. However, in practical application scenarios, there are many complex and highly uncertain factors, such as extreme weather changes, large scale and span of the target, complex background interference, motion ambiguity, etc., which makes accurate and real-time UAV target detection still a great challenge. In order to reduce the interference of these situations in real detection scenes and improve the accuracy of UAV detection, a Global Edge Information Enhance (GEIE)module is proposed in this paper, which enables edge information to be fused into features extracted at various scales. It can improve the attention of the network to the edge information of the object. In addition, special weather conditions can greatly reduce the detection accuracy of the target, this paper proposes a Multiscale Edge Feature Enhance(MEFE) module to extract features from different scales and highlight edge information, which can improve the model’s perception of multi-scale features. Finally, we propose a Lightweight layered Shared Convolutional BN(LLSCB) Detection Head based on LSCD, so that the detection heads share the convolutional layer, and the BN is calculated independently, which improves the detection accuracy and reduces the number of parameters. A high performance YOLO detector (YOLO-GML) based on YOLO11 model is proposed. Experimental results show that Compared with YOLO11s, YOLO-GML can improve AP50 by 2.3% to 73.6% on the challenging UAV detection dataset HazyDet, achieving a better balance between accuracy and inference efficiency compared to the most advanced detection algorithms. YOLO-GML also showed good performance improvement in the SODA-A and VisDrone-2019 datasets, demonstrating the generalization of the model.

## Introduction

In recent years, the application of drones in various fields has become increasingly extensive, especially in rescue and rescue, police investigation, agricultural monitoring and traffic management, and has played an important role. However, real-time detection and precise positioning are particularly difficult because the targets are often small, shooting angles vary, and weather environments are complex [1]. With its excellent generalization ability and high precision, deep learning is gradually replacing traditional algorithms and becoming the mainstream technology of UAV target detection. Therefore, it is very important to design an efficient UAV target detection algorithm [2].

Object detection models based on deep learning can be divided into two categories: single-stage detection model and two-stage detection model. Single-stage detection models such as RetinaNet, SSD series, YOLO series, EfficientDet and DETR, which generate detection boxes through direct regression to quickly identify and locate targets [3]. In contrast, two-stage detection models, such as R-CNN, Faster R-CNN, Mask R-CNN, etc., adopt the method of first generating candidate regions and then accurately locating these regions, which can usually provide higher detection accuracy [4]. Although two-stage models excel in accuracy, they are computation-based and slow to reason, which makes them less suitable for running on devices with limited computing resources, especially platforms like drones. The single-stage model greatly improves the inference speed and frame rate by means of direct regression box, so they are more suitable for deployment on UAV equipment requiring efficient operation [5]. Such high efficiency and fast response make single-stage detection model become the mainstream choice in current UAV target detection applications. Recently, improving the detection accuracy of UAVs for targets in complex environments has become a focus of research by many scholars [6]. By improving the image processing method, Bai et al. increased the proportion of small objects in the image. They used image slicing technology to separate small targets from the background, thus improving the detection algorithm’s attention to these targets. At the same time, they use the intersection ratio loss function based on the minimum point distance to optimize the boundary box matching, which improves the accuracy and robustness of the detection, so that the detection performance of the model for small targets is effectively enhanced [7]. Peng et al [8]. further improved the representation ability of multi-scale feature maps by introducing contextual semantic enhancement module. In this way, they enhanced the model’s ability to recognize targets at different scales, especially in the detection of small targets. However, Peng et al. ‘s method has a high false detection rate when dealing with similar targets, and although the overall performance has been enhanced, there are still some errors in some complex scenarios. Zhai et al [9]. focused on the fine-grained information loss caused by step-size roll-up and pooling layers. In the feature extraction stage, they propose to replace traditional convolution (Conv) with SPD-Conv to extract richer multi-scale features. In addition, they introduced the GAM attention mechanism in the neck part of the model, which effectively enhanced the fusion ability of target features, making the model more accurate when dealing with complex scenes. Li et al [10]. proposed a CEM method for the Carafe upsampling operator, which alleviated the influence of channel compression and improved the expression ability of the model. At the same time, they propose an SPM method based on the CA attention mechanism, which further enhances the model’s ability to learn the location features of interest. This series of improvements make the model more efficient in dealing with details and complex scenes, and improve the accuracy of small target detection. By proposing channel feature partial convolution (CFPConv) and reconstructed CFP_C2f modules, Fu et al [11]. have enhanced the capability of multi-scale feature extraction, especially in the capture of fine-grained targets. They embedded a context aggregation module (CAM) in the model to further enhance the detail perception of deep features, allowing the model to better understand complex information in the image.

Although the previous work has carried out multi-angle optimization of UAV target detection, due to the complexity and particularity of the image from the UAV perspective, the target is small and undistributed, and the weather environment is complex and changeable, there are still serious cases of missed detection and false detection [12]. This paper focuses on the improvement and enhancement of the backbone network, neck and detection head of the YOLO11s model from the perspective of detection accuracy of small targets in complex environments, generalization of different scenes of the model and detection speed. A new detection model, YOLO-GML, with high detection accuracy, strong generalization ability and fast detection speed, is proposed [13]. The main contributions of this paper include:

(1) Considering the problems of small UAV targets, mutual occlusion and uneven distribution, a MutilScale Edge Feature Enhance module is proposed for multi-scale feature extraction, edge information enhancement and convolution operation, and the C3k2 module is improved based on this module [14].
(2) Aiming at the problem that the environment is complex and changeable, which greatly interferes with the detection accuracy of UAV, and there is no component in the conventional target detection network that can improve the attention of the network to the Edge Information of objects, we propose a module named Global Edge Information Enhance Module. Edge information can be fused into features extracted at various scales [15].
(3) Although the detection accuracy has been greatly improved after improving the above two points, the complexity of the model has also increased. Based on LSCD, we proposed LLSCB to reduce the loss of accuracy as much as possible by using shared convolution to reduce the number of parameters and the amount of computation in the detection head [16].
(4) A new unmanned aerial vehicle detection model (YOLO-GML) in complex environment is proposed. Compared with the most advanced methods, it can achieve a better multi-directional trade-off [17].

## Related works

In the field of UAV image target detection, researchers have proposed a variety of improvement methods for different technical difficulties. Based on YOLOv8, Wang et al. introduced a global spatial attention mechanism to enhance the feature extraction capability of small targets, but faced with the problem of high false detection rate of similar targets. Zhu’s team focused on the YOLOv5 framework and replaced the traditional convolution structure with Transformer detection head and added detection head to improve the positioning accuracy of UAV aerial photography targets [18]. Xiong and other researchers proposed an adaptive feature fusion module, which integrated deep semantic and shallow spatial features to achieve multi-scale optimization, but this method affected the deployment efficiency due to module complexity [19]. Guo et al. replaced the C2f structure in YOLOv8 with a C3D module to enhance information integrity, but the computational cost increased. In addition, Zhao team adopts clustering method to optimize anchor frame parameters and improve focus loss function based on RetinaNet framework, but there are limitations in real-time performance [20]. Although the above studies have significantly improved the detection performance of the YOLO series in the UAV scene, the improvement exploration of the latest YOLO11 architecture is still relatively lacking, and its potential in complex aerial photography environments has not been fully tapped [21].

YOLO11, a new generation real-time inspection framework launched by Ultralytics, has achieved breakthrough innovation in architecture design and function expansion. The model constructs a more efficient visual perception system by systematically reconstructing network components and algorithm flow. Through the joint optimization of backbone network and feature fusion module, YOLO11 builds a hierarchical feature representation system. Its innovative C3K2 composite module adopts a heterogeneous convolution kernel combination strategy (3 × 3, 5 × 5 and other multi-scale sensing units), and realizes multi-granularity information fusion through dual-branch feature processing architecture: the main branch retains the original feature transfer path, and the auxiliary branch enhances semantic representation through deep separable convolution stack. This dial-and-conquer strategy not only expands the effective sensing field, but also maintains the computational efficiency through the dynamic feature compression of 1 × 1 convolution, which significantly improves the identification ability of large-scale targets and fuzzy boundaries in complex scenes. The innovative design of the C2PSA module deeply integrates the pyramid slice attention mechanism with the residual structure. The module constructs the spatial and channel dual attention weight through multi-level feature slice recombination, inherits the channel calibration advantages of SE module, and introduces the long-range dependency modeling across spatial domains. In particular, the nested application of Transformer structure in PSABlock enables the network to dynamically focus on key feature areas, effectively alleviating detection interference caused by target occlusion and dense alignment. Experiments show that this hybrid attention mechanism has significant gain for small target group detection in aerial images. YOLO11 realizes the precision-efficiency co-evolution through the deeply compressed modular design: In the COCO benchmark test, YOLO11m achieves a higher mAP index with 22% parameter reduction than YOLOv8m, and its dynamic calculation allocation strategy can automatically adjust the calculation chart structure according to hardware characteristics. The specially designed lightweight deployment interface supports cross-platform migration from Jetson series edge devices to cloud GPU clusters, and works with a unified multi-task framework (covering instance segmentation, pose estimation, OBB directional detection, etc.) to form a full-stack vision solution. These innovations enable YOLO11 to show stronger robustness to typical challenges such as low-altitude targets, dense scenes and motion blur from the UAV perspective while maintaining real-time performance, and provide a better technical base for application scenarios such as intelligent inspection and dynamic monitoring [22].

### Methodology

Ethical review and approval were waived for this study, due to the fact that we have implemented privacy protection for the volunteers in the images.

#### Overall of network.

**Fig 1:**
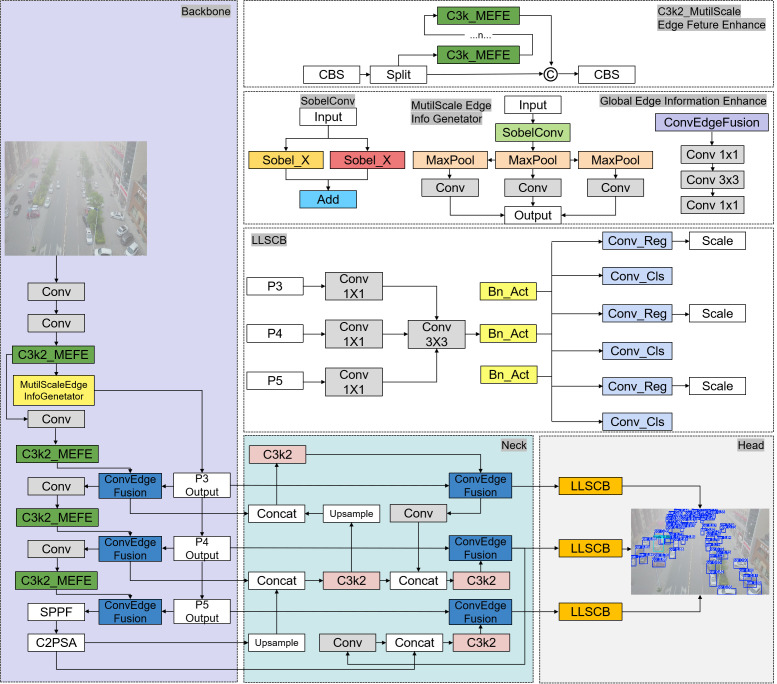
Overall structure of YOLO-GML.

Taking YOLO11s as our basic model, YOLO11 is the UAV target detector with the best comprehensive performance at present, with high accuracy and speed. Proposed GEIE, C3k2_MEFE, and LSCSBD, based on these, we proposed the YOLO-GML model, the overall structure diagram is shown in Fig 1.

YOLO-GML consists of three main components: backbone, neck, and head. In the backbone, we first introduced MutilScale Edge Feature Enhance module to generate multi-scale edge information feature maps using the shallow feature layer of the network and put them into each scale of the backbone for fusion. Second, a ConvEdgeFusion module is introduced that outputs through layers P3, P4, and P5 to help the model better integrate features from different sources. The C3k2_MEFE module is used to replace the original C3k2 module and extract features from different scales to highlight edge information. In the neck, we similarly introduce a ConvEdgeFusion module to further extract the merged features to enhance the model’s ability to capture local detail. In the head, we replace the original detection head of the model with the LLSCB detection head, so that the detection head can reduce the loss of accuracy as much as possible under the condition of fewer parameters and less calculation [23].

#### C3k2-mutilscale edge feature enhance module.

Traditional YOLO11 architectures face significant challenges in accurately detecting objects in complex environments, particularly in UAV aerial imagery captured during adverse weather conditions like dense fog [24]. This paper proposes a novel enhancement to the YOLO11 backbone by replacing the conventional C3k2 module with our C3k2_MutilScale Edge Feature Enhance module. Our approach specifically addresses the reduced contrast and blurred boundaries prevalent in foggy conditions by enhancing edge information across multiple scales [25].

When UAV images are taken in adverse weather conditions (especially fog), the performance of object detection algorithms will be significantly degraded due to the reduced contrast between the object and the background, blurred boundaries that make it difficult to depict the object, and the loss of fine-grained texture details [26].

The C3k2 module in YOLO11 architecture, while effective in standard conditions, fails to adequately address these challenges in complex environments. The module’s conventional convolutional operations do not specifically enhance edge information, which becomes particularly problematic in foggy conditions where edges are already weakened.

C3k2_MutilScale Edge Feature Enhance is introduced, and a new enhancement of C3k2 module, edge extraction and enhanced through the clear information, capture the context information in multiple scales and the adaptive fusion enhancement features as the original, It significantly improves object detection performance in challenging visibility conditions. When applied to uav image capture foggy weather conditions, the enhanced c3k2_MutilScale Edge Feature Enhance module has significantly increased than the traditional C3k2 YOLO11 module. The first is that the explicit enhancement of edge information makes object localization more accurate, especially for objects with fuzzy boundaries in foggy conditions. Secondly, the multi-scale method is used to capture the context information of different scales, which improves the ability of the network to distinguish similar objects in low contrast environments. Despite the added edge processing, our module maintains comparable computational efficiency to the original C3k2 module, making it suitable for real-time applications. The adaptive nature of the proposed method shows consistent performance in different levels of fog density, and it is more robust to changing environmental conditions.

**Fig 2:**
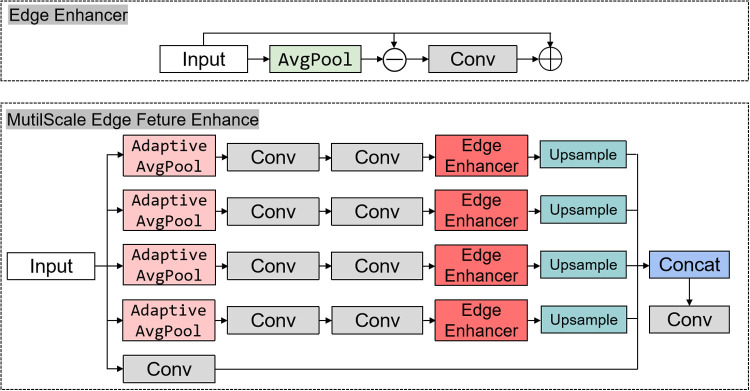
MutilScale Edge Feature Enhance module structure.

**Fig 3:**
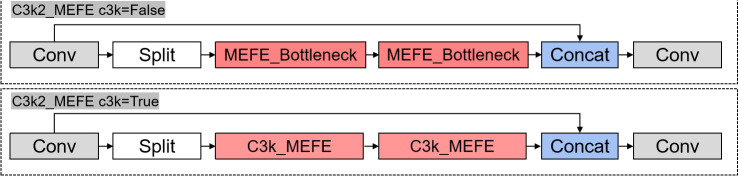
C3k2_MEFE module structure.

The structure diagram of MEFE module and C3k2_MEFE module is shown in Figs 2 and 3.

In the following, I will explain the operation logic of the module in detail. The EdgeEnhancer module is designed to explicitly enhance high-frequency edge information in the feature maps. It operates through the following steps.

Apply average pooling to extract low-frequency information:


$${\text{F}}_{\text{low}}=\text{AvgPool}\left({\text{F}}_{\text{in}}\right)$$ (1)

Subtract low-frequency information from the input to obtain edge information:


$${\text{F}}_{\text{edge}}={\text{F}}_{\text{in}}-{\text{F}}_{\text{low}}$$ (2)

Process the edge information through a convolutional layer with sigmoid activation:


$${\text{F}}_{\text{edge}}^{\prime }=\sigma ({\text{Conv(F}}_{\text{edge}})$$ (3)

Add the processed edge information back to the original input:


$${\text{F}}_{\text{out}}={\text{F}}_{\text{in}}+{\text{F}}_{\text{edge}}^{\prime }$$ (4)

This operation can be formalized as:


$${\text{F}}_{\text{out}}={\text{F}}_{\text{in}}+\sigma \left(\text{Conv}\left({\text{F}}_{\text{in}}-\text{AvgPool}\left({\text{F}}_{\text{in}}\right)\right)\right)$$ (5)

The sigmoid activation ensures that the edge enhancement is applied proportionally to the significance of the edge features, preventing over-enhancement of noise.

The MutilScale Edge Feature Enhance module extends the edge enhancement concept by incorporating multi-scale contextual information. Extract local features through a 3 × 3 convolution:


$${\text{F}}_{\text{local}}={\text{Conv}}_{3\times 3}\left({\text{F}}_{\text{in}}\right)$$ (6)

Generate multi-scale features through adaptive average pooling at different bin sizes:


$${\text{F}}_{{\text{bin}}_{\text{i}}}={\text{Conv}}_{3\times 3}\left({\text{Conv}}_{1\times 1}\left({\text{AdaptiveAvgPool}}_{{\text{bin}}_{\text{i}}}\left({\text{F}}_{\text{in}}\right)\right)\right)$$ (7)

Apply EdgeEnhancer to each scale-specific feature:


$${\text{F}}_{{\text{bin}}_{\text{i}}}^{\prime }=\text{EdgeEnhancer}\left({\text{F}}_{{\text{bin}}_{\text{i}}}\right)$$ (8)

Upsample each enhanced scale-specific feature to the original resolution:


$${\text{F}}_{{\text{bin}}_{\text{i}}}^{\prime \prime }=\text{Upsample}\left({\text{F}}_{{\text{bin}}_{\text{i}}}^{\prime }\right)$$ (9)

Concatenate local features with all enhanced multi-scale features:


$${\text{F}}_{\text{Concat}}=\text{Concat}\left(\left[{\text{F}}_{\text{local}},{\text{F}}_{{\text{bin}}_{1}}^{\prime \prime },{\text{F}}_{{\text{bin}}_{2}}^{\prime \prime },{\text{F}}_{{\text{bin}}_{3}}^{\prime \prime },{\text{F}}_{{\text{bin}}_{4}}^{\prime \prime }\right]\right)$$ (10)

Apply a final convolution to fuse all features:


$${\text{F}}_{\text{out}}=\text{Conv}\left({\text{F}}_{\text{concat}}\right)$$ (11)

This multi-scale approach allows the module to capture edge information at various scales, which is particularly beneficial for detecting objects of different sizes in UAV aerial imagery.

The C3k2_MutilScale Edge Feature Enhance module integrates the MutilScale Edge Feature Enhance module into the C3k2 structure of YOLO11. The module maintains the cross-stage partial network structure of C3k2, It replaces the conventional convolution blocks with our MutilScale Edge Feature Enhance module. For cases where c3k=True, it utilizes a more complex C3k_MutilScale Edge Feature Enhance structure. This integration enables the YOLO11 backbone to better process edge information at multiple scales without significantly increasing computational complexity.

#### Lightweight layered shared convolutional BN detection head.

Lightweight Shared Convolutional Detection (LSCD) Head significantly reduces the number of parameters in the model by adopting a shared convolutional approach. LSCD introduces the Scale layer, which aims to scale the feature maps of different levels. This strategy can effectively adapt to the needs of multi-scale target detection, so as to improve the detection accuracy and model adaptability. Although shared convolution is used to reduce the number of model parameters, there are still differences in statistical properties between features at different levels. In order to maintain the stability and accuracy of the features, the Normalization layer remains essential in this context. Traditionally, if the batch normalization (BN) is directly introduced into the detection head of shared parameters, it will cause errors in the calculation of the sliding average during training, which will affect the performance of the model. At the same time, although the use of group normalization (GN) can alleviate some problems, its introduction will increase the computational overhead in the inference stage, thus affecting the efficiency of the model. To overcome these problems, we borrowed the method of NASFPN (Neural Architecture Search Feature Pyramid Networks). A Lightweight layered Shared Convolutional BN(LLSCB)Detection Head is proposed, and a more efficient solution is adopted. In LLSCB, although convolution layers are shared between detection heads, BN operations are no longer handled uniformly, but BN is computed separately for each detection head. This approach can effectively avoid the problem of sliding average error, and avoid the extra inference cost caused by GN, so that the accuracy and efficiency of the model are taken into account.

**Fig 4:**
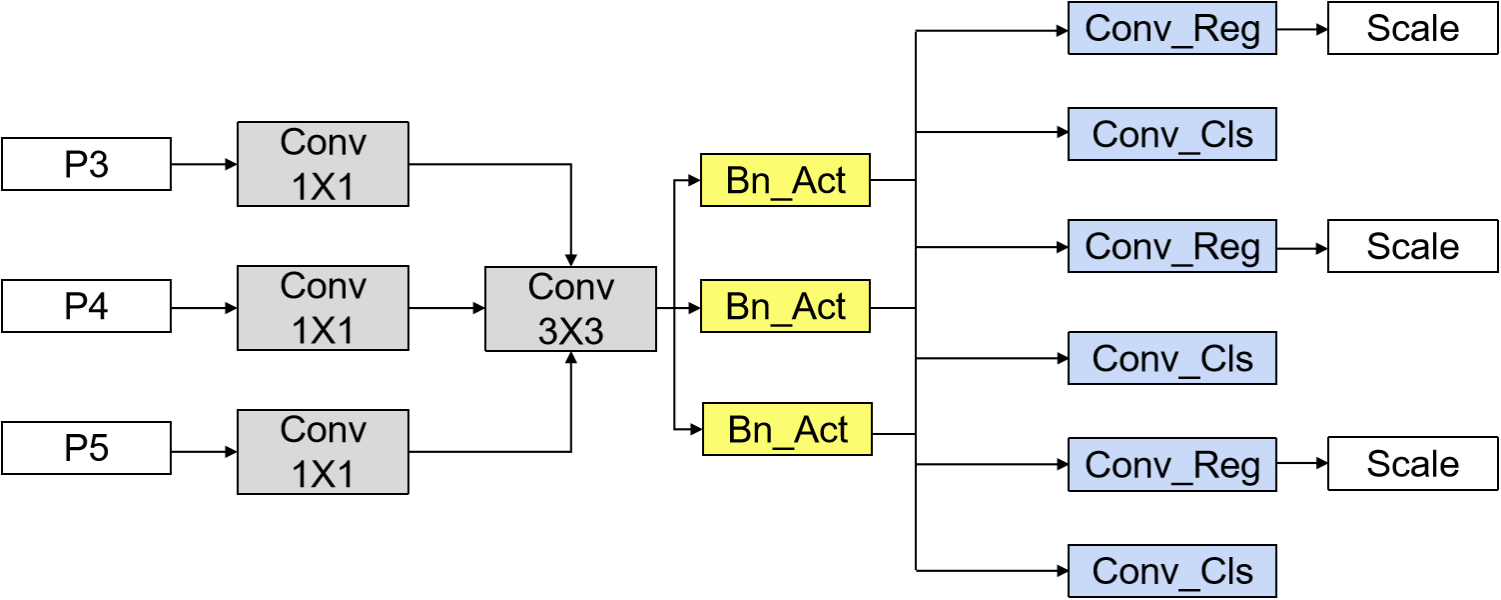
LLSCB structure.

The LLSCB employs an innovative architecture that adeptly processes feature maps from various scales, specifically P3, P4, and P5. By integrating features from these diverse scales, the model effectively synthesizes multiple levels of multi-grained information, thereby enhancing its capability to capture both the intricate details and contextual elements of objects. This approach to multi-scale feature fusion allows the model to simultaneously analyze fine-grained local features alongside coarse-grained global information, which contributes to improved precision and robustness in key point localization tasks. Furthermore, the LLSCB module significantly minimizes the number of parameters requiring training by utilizing shared convolutional parameters. This strategy not only reduces the model’s complexity but also markedly enhances computational efficiency. The design objective of the shared convolutional layer is to preserve model efficiency while ensuring real-time performance, enabling the LLSCB to deliver high performance in practical applications, particularly in environments with constrained computational resources, while maintaining rapid inference speeds. The structural representation of the LLSCB is illustrated in Fig 4.

In the LLSCB module, the processing of key point features is executed through a sequence of specifically designed convolutional layers, which consist of a 1 × 1 convolution layer, a 3 × 3 convolution layer, and a subsequent 1 × 1 convolution layer. These convolutional layers are strategically configured to effectively process feature mappings at varying scales and to extract precise key-point features. The initial 1 × 1 convolution layer serves to modify the number of channels within the feature map, thereby optimizing the flow of information. The 3 × 3 convolution layer is instrumental in capturing spatial information within local regions, enhancing the model’s ability to perceive details across different scales. The concluding 1 × 1 convolution layer ensures that the output feature channels align with the model’s specifications.

To address the potential issues associated with shared convolutional layers during batch normalization (BN) operations, we compute the BN independently for each convolutional layer rather than employing a shared BN approach. This methodology effectively mitigates the moving average error that can arise from shared BN and reduces the statistical deviation linked to parameter sharing throughout the training process. Consequently, this independent calculation of BN enhances accuracy, significantly decreases the number of parameters within the model, and improves computational efficiency.

Furthermore, to tackle the challenges encountered by each detector head when addressing targets of varying scales, the LLSCB module incorporates a scale layer designed to adjust the scale of features. This scale layer autonomously modifies the feature map in accordance with the dimensions of different targets, ensuring that each detection head can adapt flexibly to targets of various sizes while maintaining high positioning accuracy and recognition performance in the detection of both small and large targets. Through this comprehensive design, the LLSCB module not only enhances detection accuracy but also substantially improves the computational efficiency and scalability of the model.

In the LLSCB model, three feature layers generated from the neck are directed to the detection head for further processing. Initially, each branch modifies the number of channels in the input feature layer through a 1 × 1 convolution layer, thereby ensuring that all input feature layers are standardized to a fixed number of channels in the intermediate layer prior to proceeding to the subsequent stage. Subsequently, these adjusted feature layers are consolidated into a shared convolutional module, which facilitates additional feature extraction. This shared convolutional module employs a 3 × 3 convolution kernel, which is adept at capturing the spatial information of the local region while simultaneously minimizing the number of parameters and the computational demands of the model. The implementation of shared convolution not only streamlines the model’s complexity but also significantly enhances computational efficiency.

Upon the completion of feature extraction, the regression and classification branches are processed independently, each fulfilling distinct functions. To address the issue of scale inconsistencies that detection heads may face when identifying objects of varying sizes, the LLSCB employs a novel approach to modify the output of the regression branch. This is achieved through the implementation of a scale layer, which effectively adjusts the features and calibrates the output feature map of each detection head. Consequently, the regression branch is better equipped to accurately locate and regress targets of different dimensions. This strategy ensures that the model retains high levels of accuracy and robustness when confronted with objects of diverse scales.

#### Global edge information enhance module.

The traditional YOLO11 architecture has demonstrated effectiveness in general object detection tasks. However, when applied to complex UAV imaging scenarios, several inherent limitations emerge that significantly impact detection performance. The feature extraction process in the original YOLO11 backbone relies primarily on hierarchical convolutional operations that progressively downscale spatial dimensions while increasing feature channels. This conventional approach presents three key limitations when detecting objects in UAV imagery:

Edge Information Loss: The repeated downsampling operations in the traditional backbone lead to substantial loss of fine-grained edge information, which is crucial for accurately identifying small objects and precise boundary delineation in UAV imagery. This edge attenuation becomes particularly problematic in complex environments with cluttered backgrounds, variable lighting conditions, and diverse object scales characteristic of aerial surveillance [27].

Limited Multi-scale Feature Interaction: While the original YOLO11 architecture employs feature pyramid networks (FPN) for multi-scale feature fusion, the interaction between different resolution features remains constrained by the sequential processing flow. Edge features detected at different scales are not effectively propagated throughout the network, limiting the model’s ability to leverage boundary information across feature hierarchies.

Insufficient Context-Edge Integration: Traditional architectures inadequately integrate global edge context with local feature representations. In UAV imagery, where objects often appear against complex backgrounds with similar textures, the ability to precisely distinguish object boundaries from environmental edges becomes critical for reducing false positives and improving localization accuracy [28].

**Fig 5:**
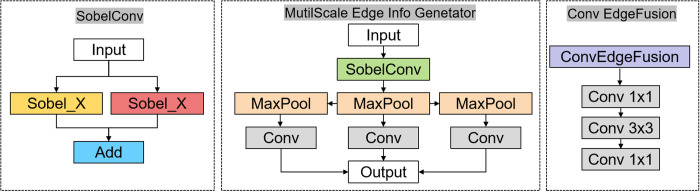
GEIE structure.

To address these limitations, we propose the Global Edge Information Enhance(GEIE) Module, designed specifically to preserve and enhance edge information throughout the feature extraction process, facilitating more effective detection of objects in challenging UAV imaging scenarios. The proposed Global Edge Information Enhance Module systematically extracts, processes, and integrates edge information across multiple scales to enhance feature representation for object detection. Fig 5 illustrates the overall architecture of our proposed module integrated within the YOLO11 framework.

The first component of our GEIE is the Multi-scale Edge Information Generator, which extracts edge features from input feature maps using Sobel operators and generates edge representations at multiple scales. The edge extraction process is formulated as:


$${\text{E}}_{\text{init}}=\text{SobelConv}(\text{X})$$ (12)

Where $x\in R^{C\times H\times W}$ is the input feature map, and SobelConv applies Sobel operators to extract gradient information. The Sobel convolution operation can be expressed as:


$$\text{SobelConv}(\text{X})=\sqrt{{\left({\text{G}}_{\text{x}}*\text{X}\right)}^{2}+{\left({\text{G}}_{\text{y}}*\text{X}\right)}^{2}}$$ (13)

Where $G_{x}$ and $G_{y}$ represent the horizontal and vertical Sobel kernels respectively, and * denotes the convolution operation. In our implementation, we utilize 3D convolution with fixed Sobel kernels to extract gradient features while preserving channel information:


$${\text{G}}_{\text{x}}={\left[\begin{array}{ccc} 1 & 2 & 1\\ 0 & 0 & 0\\ -1 & -2 & -1 \end{array}\right]}^{\text{T}},\;{\text{G}}_{\text{y}}=[\begin{array}{ccc} 1 & 2 & 1\\ 0 & 0 & 0\\ -1 & -2 & -1 \end{array}]$$ (14)

To generate multi-scale edge information, we apply sequential max-pooling operations to the initial edge map, followed by channel-wise projections using 1 × 1 convolutions:


$${\text{E}}_{\text{i}}={\text{Conv}}_{1\times 1}^{\text{i}}\left(\text{MaxPoo}l\left({\text{E}}_{\text{init}}\right)\right),\;\text{i}=1,2,...,\text{n}$$ (15)

Where $E_{i}$ represents the edge information at the i-th scale, and $Conv_{1\times 1}^{i}$ denotes the 1 × 1 convolution operation that maps the edge features to the desired channel dimension for each scale.

The second key component of our GEIE is the Edge-Feature Fusion mechanism, implemented through the ConvEdgeFusion module. This module integrates the multi-scale edge information with corresponding feature maps at different levels of the backbone network. The fusion process begins by concatenating the edge features $E_{i}$ with the corresponding backbone features $F_{i}$ along the channel dimension:


$${\text{X}}_{\text{cat}}=\text{Concat}\left({\text{E}}_{\text{i}},{\text{F}}_{\text{i}}\right)$$ (16)

The concatenated features are then processed through a sequence of convolutions to facilitate effective integration:


$${\text{X}}_{\text{fused}}={\text{Conv}}_{1\times 1}\left({\text{Conv}}_{3\times 3}\left({\text{Conv}}_{1\times 1}\left({\text{X}}_{\text{cat}}\right)\right)\right)$$ (17)

This multi-step convolution process can be formulated as:


$$\begin{array}{c} {\text{X}}_{\text{mid}}=\delta \left(\text{BN}\left({\text{Conv}}_{1\times 1}\left({\text{X}}_{\text{cat}}\right)\right)\right)\\ {\text{X}}_{\text{feat}}=\delta \left(\text{BN}\left({\text{Conv}}_{3\times 3}\left({\text{X}}_{\text{mid}}\right)\right)\right)\\ {\text{X}}_{\text{fused}}=\delta \left(\text{BN}\left({\text{Conv}}_{1\times 1}\left({\text{X}}_{\text{feat}}\right)\right)\right) \end{array}$$ (18)

Where δ represents the activation function,BN denotes batch normalization, and $Conv_{k\times k}$ indicates a convolution operation with kernel size k.

Our GEIE incorporates edge information not only in the backbone feature extraction pathway but also in the detection head, creating a comprehensive edge-aware detection framework. The edge features generated by the Multi-scale Edge Information Generator are shared across both the backbone and detection head, enabling global edge information propagation throughout the network.

This global propagation mechanism allows edge features detected at early stages to influence feature extraction and object detection at later stages, facilitating more accurate boundary delineation and reducing false positives in complex environments. The mathematical formulation for the entire GEIE-enhanced backbone can be expressed as:


$$\begin{array}{c} \{{\text{E}}_{1},{\text{E}}_{2},{\text{E}}_{3}\}=\text{MutilScaleEdgeInfoGenerator}\left({\text{F}}_{\text{P}2/4}\right)\\ {\text{F}}_{\text{P}3/8}^{\text{enh}}=\text{ConvEdgeFusion}\left({\text{F}}_{\text{P}3/8},{\text{E}}_{1}\right)\\ {\text{F}}_{\text{P4}/16}^{\text{enh}}=\text{ConvEdgeFusion}\left({\text{F}}_{\text{P}4/16},{\text{E}}_{2}\right)\\ {\text{F}}_{\text{P}5/32}^{\text{enh}}=\text{ConvEdgeFusion}\left({\text{F}}_{\text{P}5/32},{\text{E}}_{3}\right) \end{array}$$ (19)

Where $F_{Pi/j}$ represents the feature map at scale i/j, and $F_{Pi/j}^{enh}$ denotes the edge-enhanced feature map.

The Global Edge Information Enhance Module significantly improves the detection performance of the YOLO11 architecture on UAV imagery datasets. The proposed Global Edge Information Enhance Module effectively addresses the limitations of traditional YOLO11 architecture in UAV object detection by preserving and enhancing edge information across multiple scales. The edge-aware feature representation significantly improves detection accuracy, particularly in complex environments with challenging visual conditions.

## Experiments

### Implementation details

#### Dataset.

**Fig 6:**
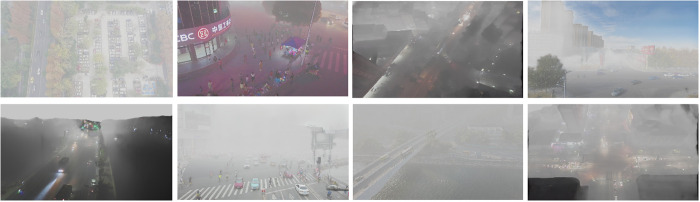
Partial images of HazyDet.

The HazyDet dataset was utilized to assess the efficacy of YOLO-GML in detecting targets from unmanned aerial vehicles (UAVs) within complex backgrounds. All models were developed from the ground up, without the use of pre-trained models. The HazyDet dataset was collaboratively established by researchers from the PLA Army Engineering University, Nankai University, Nanjing University of Posts and Telecommunications, and Nanjing University of Science and Technology. It comprises 383,000 real-world instances derived from composite images that replicate fog effects in both natural fog environments and typical scenes. This dataset presents significant challenges for drone operations under severe weather conditions and includes targets categorized into three classes: Car, Truck, and Bus. The dataset is partitioned into a training set of 8,000 images, a validation set of 1,000 images, and a test set of 2,000 images. A sample portion of the HazyDet dataset is illustrated in Fig 6.

We not only use HazyDet to evaluate the performance of our proposed algorithm, but also use the SODA-A and VisDrone-2019 datasets to demonstrate the generalization ability of our YOLO-GML in different scenarios. The images in the SODA-A dataset come from hundreds of cities around the world and have a rich data diversity. The number of image instances in the dataset ranged from 1 to 11,134, with an average of 318 instances per image, and 80,203 instances annotated in nine classes, meaning that the dataset contained not only sparse cases, but also many images where objects were located very close together. The training set contains 11837 images, the verification set contains 3309 images, and the test set contains 1507 images. The VisDrone-2019 dataset contains 288 video clips totaling 261,908 frames and 10,209 still images, covering a variety of different environments, objects and scene types. It includes images from 14 different cities in China, presenting a diverse background of urban and rural objects such as pedestrians, vehicles, bicycles, etc., with scene densities ranging from sparse to crowded. The data is collected in different weather and light conditions through a variety of drone platforms. The data set is divided into 6471 images in the training set, 548 in the verification set, and 1610 in the test set.

#### Training setting.

The experiment platform uses Ubuntu23.02 as the operating system and is equipped with 12 vCPU Intel(R) Xeon(R) Platinum 8352V CPU @ 2.10GHz. The system memory is 90GB, the graphics card is Nvidia GeForce RTX 4090, the graphics memory is 24GB, the pytorch framework version is 2.2.2 + cu121, and the python version is 3.10.14. Set the Batch size to 16, the image size to 640X640, and the epoch to 200. All the models presented in the experimental section did not use the weights of pre-trained models. All experiments were validated through the same set of hyperparameters.

#### Evaluating indicator.

In order to comprehensively evaluate the effectiveness and performance of our proposed method, we use a variety of indicators for verification, including accuracy, average average accuracy (mAP), recall rate, inference speed, and floating-point arithmetic (FLOPs). Accuracy refers to the proportion of positive samples correctly detected by the model in the total test results, which is usually used to measure the accuracy of the test results. The recall rate refers to the proportion of positive samples correctly detected by the model in all real positive samples, reflecting the detection ability of the model. In practical applications, accuracy and recall are often used to comprehensively evaluate the performance of a model, and their specific definitions are shown below:


$$\text{ Precession}=\frac{\text{TP}}{\text{TP}+\text{FP}},$$ (20)

$$\text{ Recall}=\frac{\text{TP}}{\text{TP}+\text{FN}},\;$$ (21)

TP (True cases), which is the number of samples that the model correctly classifies as positive; FP (false positive cases), that is, the number of actual negative samples that the model incorrectly classified as positive; And FN (false negative cases), which is the number of actual positive samples that the model incorrectly classified as negative. Average average accuracy (mAP) is used to quantify the overall accuracy level of the model and is calculated as follows:


$$\text{ AP}=\int_{0}^{1}\text{P}(\text{R})\text{dR},\;$$ (22)

$$\text{mAP}=\frac{1}{\text{N}}\sum_{\text{i}=1}^{\text{N}}{\text{AP}}_{\text{i}},$$ (23)

mAP@0.5 refers to the average accuracy of the model when the IoU (Intersection over Union) threshold is set to 0.5. Meanwhile, mAP@0.5:0.95 indicates that the model is evaluated under several different IoU thresholds ranging from 0.5 to 0.95 with a step size of 0.05. The accuracy under each threshold is calculated and averaged. For simplicity, we usually use AP50 and AP for mAP@0.5 and mAP@0.5:0.95, respectively.

### Comparisons

#### Comparisons on HazyDet.

The comparison results of different models on HazyDet are shown in Table 1. For the sake of data accuracy, we trained the models from scratch instead of using pre-trained models and tested their runtime on the RTX4090. YOLO-GML can achieve 53.3% AP and 73.6% AP50, which is the best performance compared to other one – and two-stage models.

**Table 1:** Comparisons of state-of-the-art detectors on HazyDet Test.

Method	AP50	AP	Precision	Recall	GFLOPs	Speed(FPS^4090^)	Para(M)
Two-stage:							
Faster-RCNN [29]	48.7	/	/	/	201.72	/	41.35
Grid-RCNN [30]	50.5	/	/	/	317.44	/	64.46
Cascade RCNN [31]	51.6	/	/	/	230.40	/	69.15
One-stage:							
YOLOV3 [32]	35.0	/	/	/	20.19	/	61.63
VFNet [33]	51.1	/	/	/	187.39	/	32.89
TOOD [34]	51.4	/	/	/	192.51	/	32.02
YOLOV5n	64.1	44.1	77.5	57.4	6.0	189.7	2.21
YOLOV5s	68.7	48.5	80.1	61.9	18.9	186.9	7.84
YOLOV8n	65.5	45.4	78.3	58.5	6.8	195.4	2.71
YOLOV8s	70.4	50.5	81.1	64.2	23.5	189.5	9.85
YOLOV10n	65.6	45.1	78.6	58.4	6.7	154.2	2.28
YOLOV10s	71.1	50.8	80.1	64.6	21.6	146.1	7.24
YOLO11n	66.6	46.1	78.5	59.4	6.5	168.5	2.61
YOLO11s	71.9	51.8	81.5	65.2	21.5	162.9	9.44
YOLO-GML(Ours)	73.6	53.3	81.9	67.0	28.7	158.3	11.66

Faster-RCNN, Grid-RCNN and Cascade RCNN are popular two-stage detection algorithms. The YOLO-GML is clearly superior to them in accuracy and other aspects. Our model achieved a 45.7% increase in AP50 compared to Grid-RCNN at GFLOPs(28.7 vs. 317.44). Compared with Cascade RCNN and Faster-RCNN, YOLO-GML is 42.6% and 51.1% ahead in AP50, and also has significant advantages in GFLOPs and Parameter.

**Fig 7:**
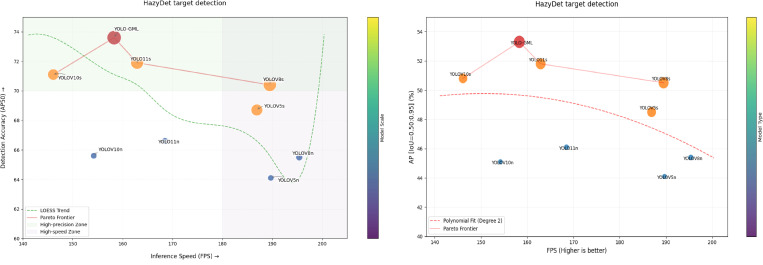
Scatter plots of AP, AP50 and FPS for the HazyDet dataset.

**Fig 8:**
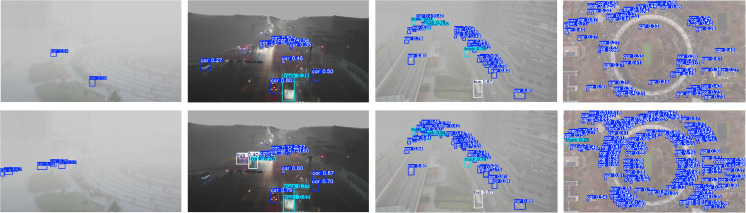
Comparison of HazyDet test results.

In addition, YOLO-GML achieves a better balance between accuracy, model size, and speed than other state-of-the-art single-stage detection algorithms. Compared with YOLOV5s, the AP50 and AP of our model are increased by 7.1% and 9.8% respectively, and the reasoning speed of loss is also in a reasonable range. Compared with YOLOV8s and YOLOV10s, the AP50 and AP of YOLO-GML increased by 4.5%, 5.5%, 3.5% and 4.9%, respectively. Compared to the baseline model YOLO11s, AP50 improved by 2.3% and AP by 2.8%, the validity of our proposed model is proved. It is relatively difficult to improve the accuracy of targets, especially small targets, in foggy weather scenes. The increase in Gflops resulting from the improved accuracy is within a completely acceptable range. In order to further show the advantages of our proposed model, we use the scatterplot to show the relationship between AP, AP50 and FPS. The result is shown in Fig 7. The comparison of some test results is shown in Fig 8. The test results of the first behavior baseline model YOLO11s and the second behavior YOLO-GML model clearly show that in extreme fog weather, our proposed model can detect more targets than YOLO11s, reducing the probability of missed detection and false detection.

#### Comparisons on different datasets.

In order to further demonstrate the effectiveness and generalization of the YOLO-GML model, we also conducted similar comparative experiments on the extremely challenging SODA-A and VisDrone-2019 data sets, and the experimental results are shown in Table 2. It can be seen that in SODA-A data set, compared with YOLO11n, AP50 and AP, the model we proposed increased by 15.7% and 23.0% respectively. Compared with the baseline model YOLO11s, AP50 increased by 1.1% and AP increased by 3.3%. It is proved that the proposed method also has high performance in the detection of very small targets and multi-targets in different scenarios.

**Table 2:** Comparisons of on SODA-A and VisDrone-2019 Test.

DataSets	Method	AP50	AP	Precision	Recall
SODA-A	YOLO11n	67.2	37.3	70.5	64.4
	YOLO11s	76.9	44.4	80.5	72.6
	YOLO-GML	77.8	45.9	80.8	72.0
VisDrone-2019	YOLOV5s	33.9	17.6	47.1	33.7
	YOLOV8s	38.3	22.9	49.7	37.5
	YOLO11s	39.0	23.4	51.1	37.6
	YOLO-GML	41.5	25.2	51.4	40.3

In VisDrone-2019 data set, compared with YOLOV8s, AP50 and AP of our model increased by 8.3% and 10.0% respectively. Compared with the baseline model YOLO11s, the AP50 improved by 6.4% and the AP improved by 7.6%. It is proved that YOLO-GML has advantages in multi-scale target detection under different illumination and different viewing angles.

**Fig 9:**
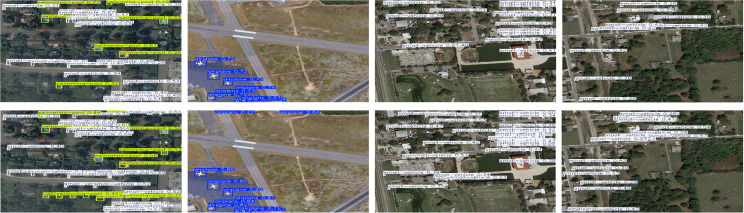
Comparison of SODA-A test results.

**Fig 10:**
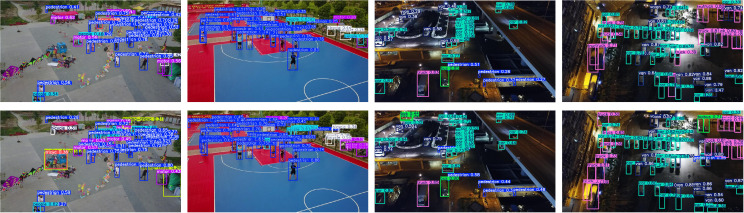
Comparison of VisDrone-2019 test results.

The test results of SODA-A are shown in Fig 9, and the test results of VisDrone-2019 are shown in Fig 10. According to the detection results of the first behavior baseline model YOLO11s and the second behavior YOLO-GML, it can be seen that in the detection of different scenarios and different targets, YOLO11s has different degrees of missed detection and false detection, while the model proposed by us has stronger detection ability.

### Ablation study

#### Ablation study of network designed.

**Fig 11:**
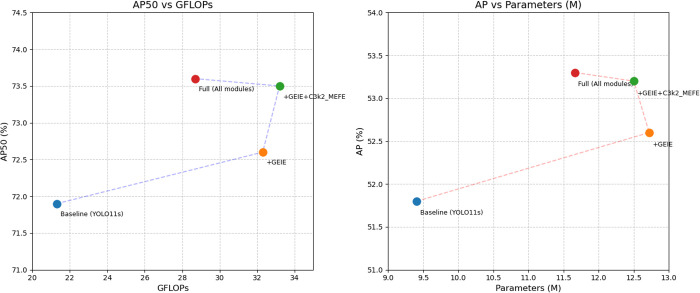
Visualization comparison chart of ablation experiments.

To prove the validity of the proposed module, we conducted ablation experiments on the three module parts mentioned in Section 3.1, using YOLO11s as the baseline model and HazyDet dataset. The performance of the different components is shown in Table 3, the more intuitive visualization comparison chart of the ablation experiments is shown in Fig 11. It can be seen that after the addition of GEIE module, AP50 and AP reach 72.6% and 52.6%, which has a small improvement compared with the baseline model, proving that this module can improve the network’s attention to the edge information of objects in complex environments. After adding GEIE and C3k2_MEFE, the AP50 and AP of the baseline model increased by 2.2% and 2.8%, which proves that this module can extract features from different scales and highlight edge information in small target scenes in foggy weather. However, GFLOPs and Parameter increase to different degrees. After joining LLSCB, AP50 and AP reach 73.6% and 53.3%, while GFLOPs and Parameter decrease to different degrees, which proves that this module can reduce the number of parameters and calculation amount by using shared convolution.

**Table 3:** Ablation study of network designed.

YOLO11s	GEIE	C3k2_MEFE	LLSCB	AP50	AP	GFLOPs	Para(M)
√				71.9	51.8	21.3	9.41
√	√			72.6	52.6	32.3	12.72
√	√	√		73.5	53.2	33.2	12.50
√	√	√	√	73.6	53.3	28.7	11.66

#### Visualizations.

**Fig 12:**
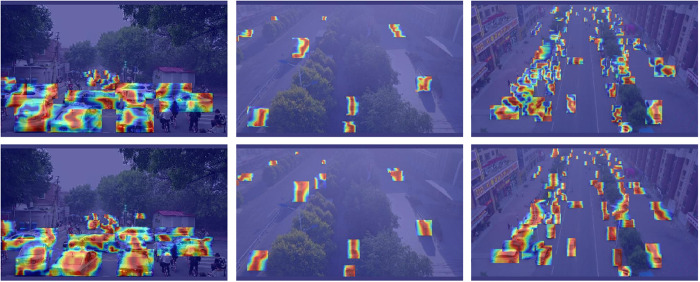
Visualization of HazyDet test results.

**Fig 13:**
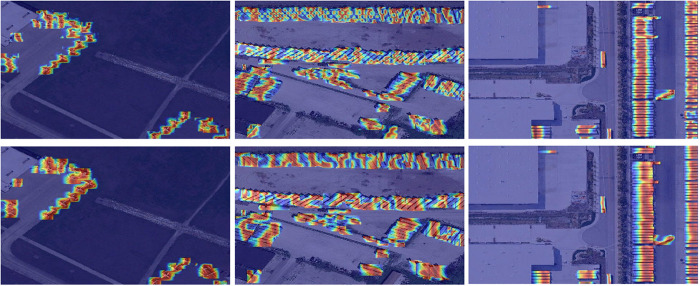
Visualization of SODA-A test results.

**Fig 14:**
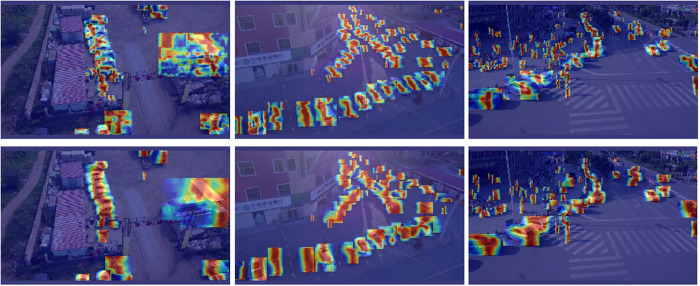
Visualization of VisDrone-2019 test results.

In order to more directly observe the enhanced effect of YOLO-GML detection, thermal maps were used for comparison, and the test results were shown in Figs 12–14. The first line in Fig 12 shows the visualization results of YOLO11s, and the results of the YOLO-GML model are shown in the second line. In the case of foggy weather, it can be seen that the detection results of the second line are smoother and more complete, indicating that the network can effectively learn key areas by focusing on the significant features of the target, and use these central information to improve the accurate identification and positioning of the target, thus reducing the uncertainty of the boundary in the decision-making process of the model.

In Fig 13, the first row of images is the detection results of SODA-A data set YOLO11s, and the second row is YOLO-GML. It can be seen that when detecting very small targets, our model pays more attention to the features of the edge of the target and pays more attention to the useful information of the target, which indicates that the proposed model has good robustness for the very small targets of UAVs. In Fig 14, the first line is the detection result of VisDrone-2019 data set by using YOLO11s, and the second line is YOLO-GML. It can be seen that in the case of different angles and scales, our proposed model can retain more details and edge information of the target, and has better detection effect than the baseline model. It is proved that YOLO-GML has a good representation ability in feature extraction and edge enhancement, which can highlight edge information and improve the attention of the network to the edge information of objects.

## Conclusion

In this paper, we propose GEIE and C3k2_MEFE to improve the accuracy of target detection in different scenarios, especially in extreme weather. The proposed GEIE helps the algorithm to transfer the edge information extracted from shallow features to the whole backbone and fuse with features of different scales. The proposed MEFE module combines multi-scale feature extraction, edge information enhancement and convolution operations to enhance the sensitivity of the network to the edge. In addition, we propose LLSCB based on LSCD, referring to the practice of NASFPN, so that the detection headers share the convolutional layer, and the BN is calculated separately. Finally, we construct a drone image detector YOLO-GML on this basis. Without pre-training, the YOLO-GML achieved 53.3 percent AP and 73.6 percent AP50 at 158 FPS, respectively. Compared to other excellent detectors (Grid-RCNN, Cascade RCNN, YOLOv8, YOLOv10 and YOLO11), a better trade-off is achieved between the detection speed and accuracy of drone images. In order to reflect the generalization of the model, we also conducted experiments in the SODA-A and VisDrone-2019 data sets, and their AP50 increased by 1.1% and 6.4% respectively compared with the benchmark model, which proved the good generalization of the model.

Although the YOLO-GML algorithm shows significant improvement in detection performance in complex scenes, there is still room for improvement for some simple scenes and specific categories of optimization effects. In the future, we will further improve the accuracy of edge information extraction in other scenes and categories and optimize the efficiency of memory access. The extensibility of the framework in multivariate detection tasks, including instance segmentation and attitude estimation, will also be verified. Finally, we will conduct explorations on the real-time performance of the improved model on edge devices with limited computing resources, aiming to enhance the model’s performance in dynamic scenarios.
